# CREATIVE ARTS INTERVENTION FOR ISOLATED LATINX OLDER ADULTS TO REDUCE TRAUMA SYMPTOMS

**DOI:** 10.1093/geroni/igad104.0141

**Published:** 2023-12-21

**Authors:** Sandy Alcantar

**Affiliations:** Long Beach Mental Health, Long Beach, California, United States

## Abstract

Creative arts is an important form of therapeutic psychotherapy to reduce trauma symptoms in migrant older adults by allowing them to explore what they once connected in their country of origin. This therapeutic modality helps to destigmatize mental health, promote health education, and feel accepted, include social connectiveness with individuals who have suffered similar trauma in their country of origin and have migrated to find a safer living environment. Interventions used have been tailored to Spanish Speaking Older adult immigrant clients that are at higher risk of mental health conditions, such as depression, anxiety, and posttraumatic stress disorder. These clients are often hiding in isolation and feel lonely. The main objective was to reduce anxiety due to re-traumatization of symptoms more importantly during pre/post COVID isolation and multiple stressors the country has been going through. For these individuals, their arts are their livelihoods due to their immigration status. It is imperative to encourage our elderly minority community to continue to focus on their crafts i.e., weaving, embroidery, sewing, crochet, cooking, pottery, or any creative arts they pr. This allows the person to have control, autonomy, feel useful and most importantly have a sense of belonging to their community, in a country that they feel displaced from. It is vital to use arts as an option to help our older adults process their life story to support development theories, in this case, Erik Erikson’s final stage of psychosocial development, integrity vs despair.

